# An update on Ym1 and its immunoregulatory role in diseases

**DOI:** 10.3389/fimmu.2022.891220

**Published:** 2022-07-28

**Authors:** Qi Kang, Luyao Li, Yucheng Pang, Wenhua Zhu, Liesu Meng

**Affiliations:** ^1^Institute of Molecular and Translational Medicine, and Department of Biochemistry and Molecular Biology, School of Basic Medical Sciences, Xi’an Jiaotong University Health Science Center, Xi’an, China; ^2^Key Laboratory of Environment and Genes Related to Diseases, Xi’an Jiaotong University, Ministry of Education, Xi’an, China; ^3^Department of Clinical Medicine, Xi’an Jiaotong University Health Science Center, Xi’an, China; ^4^National Joint Engineering Research Center of Biodiagnostics and Biotherapy, Second Affiliated Hospital, Xi’an Jiaotong University, Xi’an, China

**Keywords:** Ym1/Chil3, chitinase like protein, innate immunity, immunoregulatory roles, inflammation related diseases

## Abstract

Ym1 is a rodent-specific chitinase-like protein (CLP) lacking catalytic activity, whose cellular origins are mainly macrophages, neutrophils and other cells. Although the detailed function of Ym1 remains poorly understood, Ym1 has been generally recognized as a fundamental feature of alternative activation of macrophages in mice and hence one of the prevalent detecting targets in macrophage phenotype distinguishment. Studies have pointed out that Ym1 may have regulatory effects, which are multifaceted and even contradictory, far more than just a mere marker. Allergic lung inflammation, parasite infection, autoimmune diseases, and central nervous system diseases have been found associations with Ym1 to varying degrees. Thus, insights into Ym1’s role in diseases would help us understand the pathogenesis of different diseases and clarify the genuine roles of CLPs in mammals. This review summarizes the information on Ym1 from the gene to its expression and regulation and focuses on the association between Ym1 and diseases.

## Introduction

Ym1, known as chitinase-like protein 3 (Chil3), is a member of chitinase-like proteins (CLPs) specifically produced by rodents, which is also referred to as eosinophil chemotactic factor (ECF-L) since it was originally purified as eosinophilic crystals in mice with pulmonary inflammation. Chitinases refer to a class of chitin-degrading enzymes produced in the host, which have been proven to play a protective role in innate immunity against the chitin-containing pathogens, including parasites, fungi, and arthropods (1). CLPs lose their activity on chitin degradation due to mutations of key sites in the enzyme domain, but still participate in various inflammatory responses in mammals. Among the three CLPs in mice (Ym1, Ym2 and BRP-39), and two in humans (YKL-39 and YKL-40), YKL-40 retains the chitin-binding property, while Ym1 has a specific binding affinity for chitin-like saccharides such as glucosamine (GlcN) oligosaccharides, heparin and heparan sulfate (HS), which is presumed to belong to a new lectin family (2).

The producing cells of Ym1 are alternatively activated macrophages (Mφ) denoted as M2 or M2a (3), and neutrophils (4). It is expressed under the physiological state, and is induced by type 2 cytokines in the pathological condition, which often leads to a significant upregulation in the acute stage of inflammation. The enriched Ym1 even forms crystals in specific pathological environments (5). Ym1 has been used as a marker of M2 and is involved in the modulation of Mφ activation, the expression of Th2 cytokines and IL-17, the chemotaxis of neutrophils (and probably eosinophils) and other inflammatory responses (6, 7).

Ym1 has been confirmed to contribute to the immunopathology of certain diseases in the lung, brain, skin, joint, etc (6–8). However, the influencing factors of these diseases are complicated and diverse, and the existence of Ym1-highly-similar homologs becomes a constraint in its research techniques. Therefore, the role of Ym1 in disease pathogenesis remains unclear.

## Basics of *Chil3* gene

**Figure 1:**
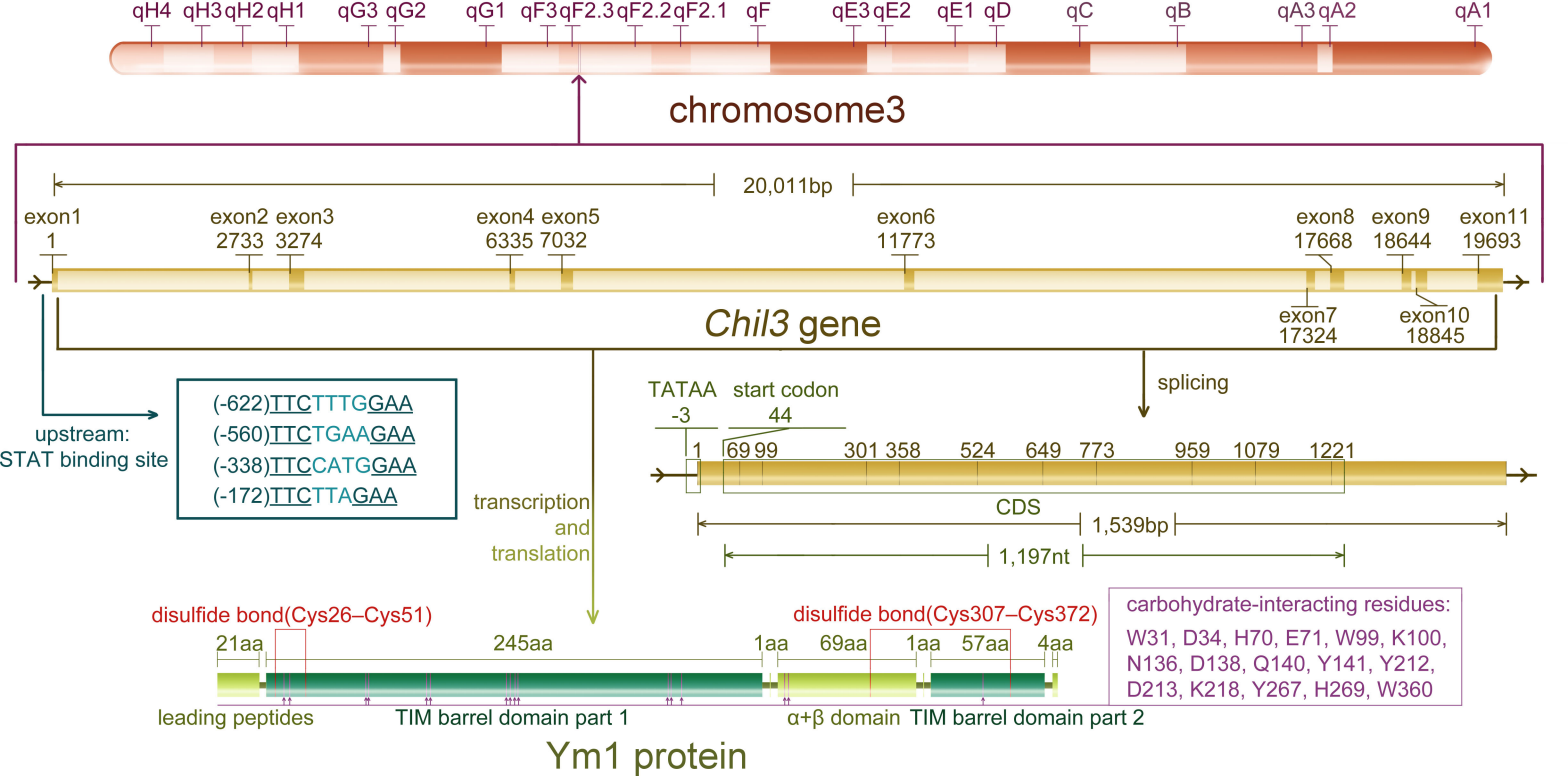
The information of *Chil3* gene, RNA and protein. *Chil3* gene is situated in the F2.2 region of mouse chromosome 3, it is composed of 20,011 pairs of bases, containing 11 exon sequences, which are 1,539bp in length. Four STAT binding sites are located near exon 1. The 1,197nt CDS sequence encodes the Ym1 protein containing 398 residues. Ym1 protein, excluding the first 21 leading peptides and the last 4 carboxyl terminal residues, can be divided into a large β/α barrel (TIM barrel) domain and a small α+β domain. Ym1 also has several carbohydrate-interacting residues though it has no chitinase activity, and these residues are marked on the diagram.

As a rodent-specific gene, *Chil3* is situated in the F2.2 region of mouse chromosome 3. As early as 1998, *Chil3* was reported to locate in the center of mouse chromosome 3, which is also an equivalent region to human chromosome 1 p13 (9). The same conclusion was also obtained by Southern blotting in 2001, indicating that *Chil3* is a single copy gene (2). What people first learned about *Chil3* was its cDNA sequence out of the mouse peritoneal exudate cDNA library, which was deposited in the GenBank database (GenBank M94584) in 1992. Subsequently, the second full-length cDNA sequence *ECF-L* was deposited in the database (GenBank D87757) in 1996. Now people know the *Chil3* gene is composed of 20,011 pairs of bases, containing 11 exon sequences, and the position of exon-intron splice site is consistent with that of human *CHI3L1* gene, but there is no splice site corresponding to the last *Chil3* splice site in human genes (9). For the transcriptional start site, 20 nucleotides upstream of the translation start codon ATG in exon 1 were determined by primer extension analysis, and now it’s generally believed that there are 43 nucleotides upstream of ATG in exon 1. Meanwhile, there are four signal transducer and activator of transcription (STAT)-binding sites TTCNxGAA near the upstream of exon 1, in which only the first and the third one could bind STAT6-containing complexes with high affinity in the EMSA experiments (10). In addition, for Ym1 protein, it was found to be a single peptide chain containing 373 residues, excluding the first 21 leading peptide and the last 4 carboxyl terminal residues, clearly divided into a large β/α barrel (TIM barrel) domain and a small α+β domain (11, 12). The information on *Chil3* gene, RNA and protein are summarized in **Figure 1**.

All gene sequences of CLPs are highly similar, in the same or different species, but there are still differences among them. Since *Chil3* gene are similar to all the known human chitinase-like genes (~ 50% nucleotide similarity), the exact human ortholog of *Chil3* cannot be clearly defined by now (9). At the same time, there is a high degree of homology between *Chil3* and *Chil4* genes in mice (9). Although the basic expression patterns of *Chil3* and *Chil4* do not overlap substantially, the first 1,200 nucleotides upstream of exon 1 of *Chil3* are 92% identical to those of *Chil4* and contain a STAT-binding site, while the first 1,700 nucleotides downstream of exon 1 of *Chil3* are 93% the same as those of *Chil4*. This high genomic similarity indicates that *Chil3* and *Chil4* are generated by relatively recent gene duplication events (10). At the evolutionary level, the research on GH18 family proteins showed that mouse CLP genes (*Chil3, Chil4, etc.*) evolved from the rodent *Chia* gene. A replication event produced CLPs, which lost its catalytic motif before further branching and expanding, may lead to the birth of *Chil3, Chil4*, and their predicted homologous pseudogenes GM6522 (previously assumed *Ym3*, *Ym4* were parsed into single predicted pseudogenes by the database) (13). The similarity of Ym1 and Ym2 once hindered the determination of the research object. At the RNA level, reverse transcription-polymerase chain reaction with specific primer pairs targeting the differences of *Chil3* and *Chil4* sequences allows the identification of the two genes (10, 14). At the protein level, Ym1 and Ym2 share a considerable number of sequences (91.7% of the amino acid sequences are the same, 33 different amino acids). In early years studies, monoclonal antibodies against Ym1 had the same effect on Ym2 (15), so immunological detection had to work in conjunction with mRNA *in situ* hybridization to make a distinction (16). However, in a recent study, specific antibodies targeting Ym1 and Ym2 respectively have been developed, and the two proteins now can be distinguished successfully (17). For early studies where Ym1 and Ym2 were not clearly distinguished, in this review we collectively refer to the relevant research objects as Ym protein.

## Ym1 expression and its regulation

**Figure 2:**
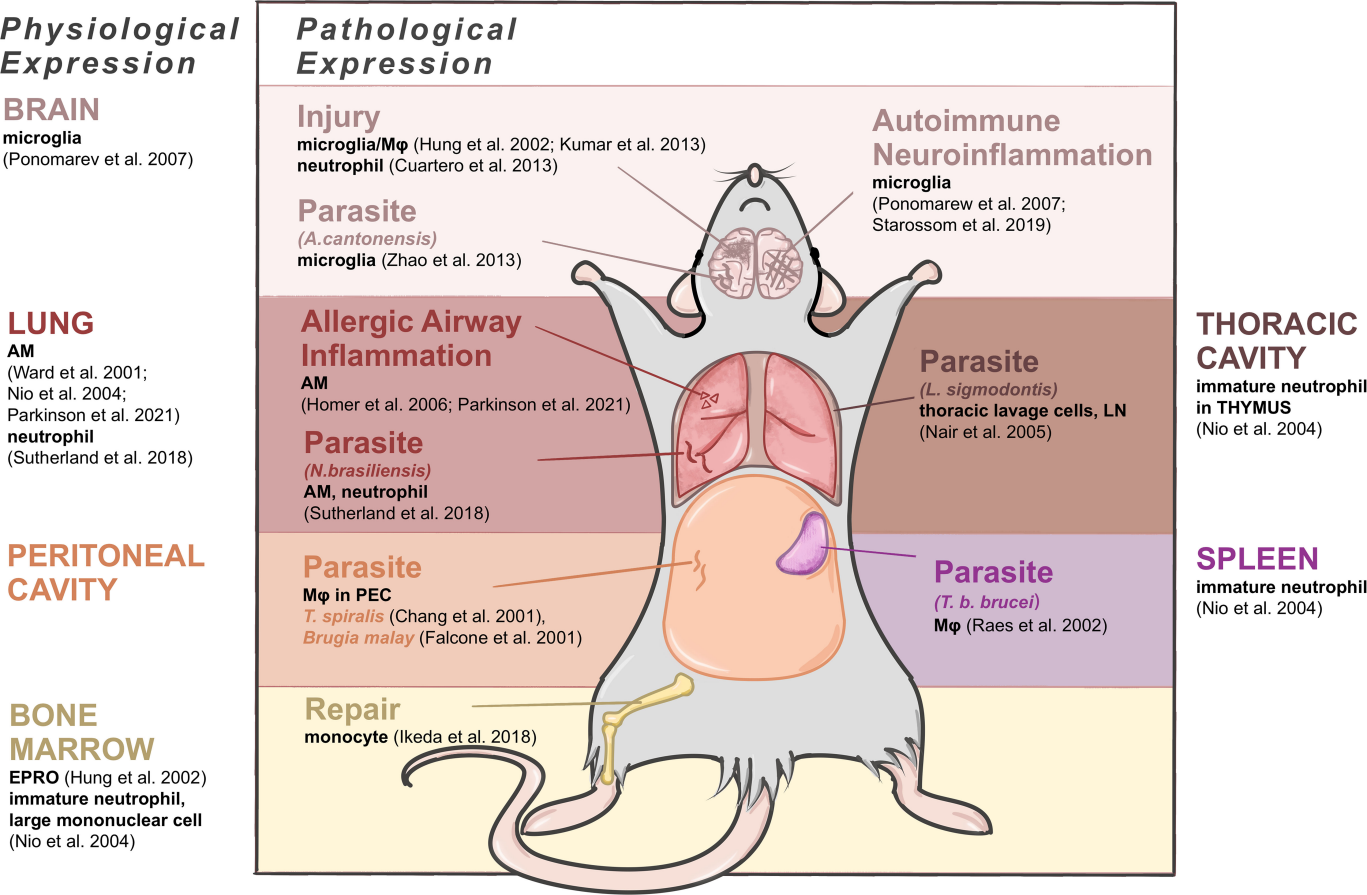
Expressing cells of Ym1 in mice under physiological and pathophysiological conditions. Brain produces Ym1 by M2-typed microglia normally, by microglia in the parasitic infection and autoimmune neuroinflammation, and by microglia, Mφ and neutrophils in injuries. Lungs express Ym1 by alveolar macrophages (AMs) and/or neutrophils in normal, allergic lung inflammation and parasitic conditions. Thoracic cavity and peritoneal cavity express Ym1 in parasite infection respectively by thoracic lavage cells and Mφ in peritoneal exudative cells. Spleen and bone marrow are also main normal origins of Ym1 performed by immature neutrophils. The former also produces increased Ym1 in Mφ during parasite infection, and the latter does it by monocytes when repairing tissue injuries.

The expression patterns of C/CLPs including Ym1 have obvious tissue specificity, thus here we try to summarize Ym1 expression accordingly under both physiological and pathophysiological conditions (as shown in **Figure 2**).

### Physiological condition

In healthy adult mice, Ym1, with different cellular origins, is constitutively expressed in the spleen, bone marrow and notably in lungs (16). Ym1 was originally purified from the supernatant of mouse splenocyte culture as an eosinophil chemokine (18), and the expressing cells were identified as immature neutrophils in red pulp (16). In the lung, alveolar macrophages (AMs) (16, 17, 19, 20) and neutrophils (4) constitutively contain Ym1. In the bone marrow, Ym1 was reported to be expressed in myeloid cells (20), and subsequent studies have further located the expressing cells as myeloid progenitor cells (destined to be monocytes or neutrophils) (19) and immature neutrophils and large mononuclear cells (16). And as neutrophil progenitors mature, their Ym1 expression will decrease to undetectable (16). Some bone marrow-derived cells, like osteoclast precursors (early immature proliferative mononuclear phagocytes) and mature osteoclasts (OCs) (21) and connective tissue type-like mast cells (22), were also detected to produce Ym1. Subsequent studies related to central and peripheral nervous system pointed out that alternatively activated microglia could also produce Ym1 in physiological condition, though at a relatively low level (23). And the accumulation of Ym1 protein in olfactory epithelia was observed during normal aging process (24).

For fetal mice, it might be noteworthy that during the development, the trace of Ym1 expression coincides with the migration of tissue resident Mφ. Ym1 is initially expressed in the yolk sac and then in the liver, spleen and bone marrow where early myeloid precursor cells in hematopoietic tissues undertake Ym1 expression, and later the expression of Ym1 in newborn mice reaches its peak in the liver and spleen (19). Similarly, the vast majority of tissue resident Mφ are derived from erythromyeloid progenitor cells (EMPs) in the yolk sac, including microglia in brain and AMs in lung, and EMPs then migrate and colonize in the newborn fetal liver as well (25). For newborn mice, Ym1 in the lung becomes detectable only about two weeks after birth, while their liver and spleen gradually decrease Ym1 production, and finally, the expression status becomes consistent with that of adult mice (19). In general, AMs and lung have a close link with Ym1 in terms of the development and physiological expression, also in terms of pathology and diseases actually, thus considerable research has been devoted to relevant fields.

### Pathophysiological condition

Ym1 can be transiently induced according to various inflammatory stimuli. Allergic airway inflammation is one of the major causes of increased Ym expression. Ym1 is produced by AM (26) and contained in bronchoalveolar lavage fluid (BALF) (15, 27). Besides, parasite response enhances pulmonary Ym1 production as a universal feature (28). A quintessential example of Ym1 response to parasites was provided by the gastrointestinal nematode *Nippostrongylus brasiliensis (N. brasiliensis)*, in which Ym1 was detected in Mφ and neutrophils of lungs mainly, accompanied by a rising secretion but a reduced cell ratio (4).

In peritoneal cavity, the expression of Ym1 mainly appears in parasite infection, related to activated Mφ in peritoneal exudative cells, such as acute-phase response caused by *T. spiralis* (2) and *Brugia malay* (29). Similarly, the infection of parasite *L. sigmodontis* in thoracic cavity could also cause thoracic lavage cells to upregulate Ym1 expression (28).

In central nervous system (CNS), its expression level is upregulated during certain phases of neurotraumatic and neurodegenerative diseases, drug-induced epilepsy, autoimmune neuroinflammation as well as parasite infection (19, 24, 30), for which it has been regarded as a significant marker of the alternatively activated microglia/Mφ. Nevertheless, the specific cellular origins and time course concerning Ym1 expression under these circumstances remain elusive. Mice infected with *Angiostrongylus cantonensis* (*A. cantonensis*) were found to synthesize Ym1 primarily *via* microglia, while the infiltrating macrophages contributed more to producing Ym1 in the stroke-injured mice (31). It is also noteworthy that neutrophils can express Ym1 in the focal cerebral ischemia mice (32). In marked contrast to the CNS, Ym1 can be secreted by the supporting cells in the olfactory epithelium, and its distribution is less confined, ranging from the injured olfactory mucosa to the dorsolateral turbinates of the nasal cavity (24).

In other tissues and organs Ym1 expression in specific situations can be found as well. After *Trypanosoma brucei brucei* (*T. b. brucei*) infection, significant expression of Ym protein was detected in splenic Mφ (3). In the draining lymph nodes after parasitic infection, antigen-presenting cells were reported as the only cell group producing Ym1, most highly in B cells and Mφ (28). In traumatic wounds, Ym1 expression was restricted to granulation tissue, closely related to neutrophils rather than Mφ (33). Most of the pathological upregulation of Ym1 mentioned above emerged in the acute stage of inflammatory injury. Bone marrow, however, was observed an augment in Ym1 expression in the repairing period of tissue injury as well, explained by the fact that precursor cells of Ly6C^hi^ monocytes differentiate and proliferate into Ym1-expressing monocytes (Ym1^+^Ly6C^hi^), which would infiltrate into corresponding injury sites (34).

In sum, under the stimulation of different pathogenic factors or in different phases of the same pathological process, the specific synthesis sites of Ym1 are different. These differences are gradually becoming crucial components of a proliferation of studies. What can be applied to various parts is that Mφ (including microglia) and neutrophils constitute the dominant Ym1 expressing cells in the pathological state.

### Regulation of Ym1 expression

**Figure 3:**
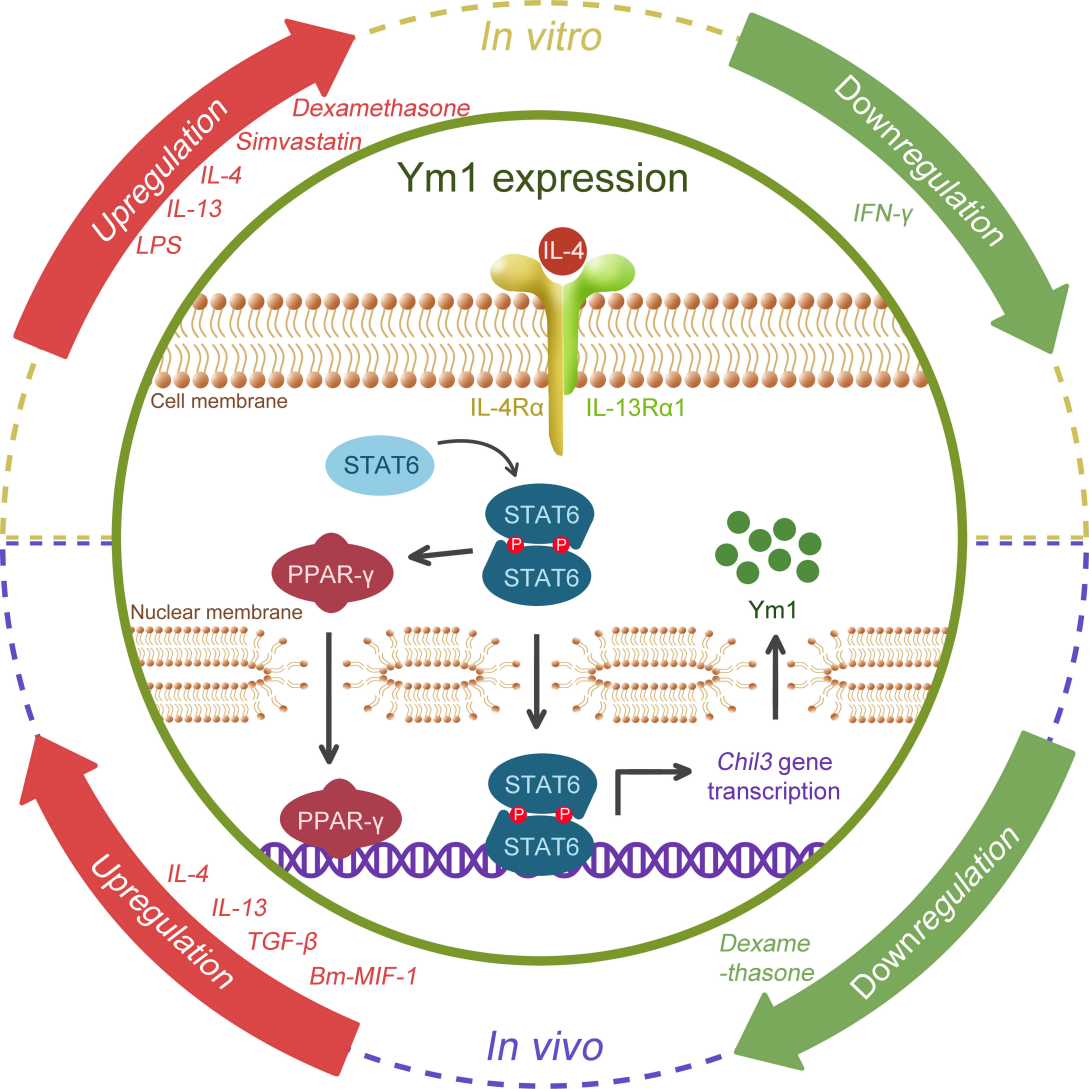
Schematic regulation of Ym1 expression. Various cytokines, drugs, microbial antigens are possible regulators. IL-4 and IL-13, the popular M2 drivers, were confirmed to promote Ym1 expression in both the *in vitro* and *in vivo* studies. TGF-β, Bm-MIF-1, LPS and Simvastatin were also found to be associated with Ym1 upregulation, and IFN-γ might correlate with its downregulation, while divergent views of dexamethasone remain in place. However, how Ym1 expression is fine-tuned by these signals remains elusive. The figure particularly illustrates the well-studied IL-4/STAT6 pathway. When stimulated by IL-4, STAT6 is phosphorylated, forming a homodimer and translocating to the nucleus, activating the gene transcription of *Chil3*. STAT6 can further activate PPAR-γ, which enhances *Chil3* expression cooperatively.

Previous studies have found that multiple inflammatory factors or stimulating drugs are able to induce or regulate the expression of Ym1 (as shown in **Figure 3**). It has been confirmed that Ym1 expression is mediated by STAT6 and induced by IL-4/IL-13 in Mφ, dendritic cells (DCs) (3, 10, 35–38) and microglia (39, 40), and Ym1 is the reigning Ym protein subtype induced by IL-4 (41). The effects of IL-4 and IL-13 are different to some extent, and IL-13 might offer a more powerful inducement on Ym1 expression *in vivo* (38). Intraperitoneal injection of anti-IL-4 antibody can block the expression of Ym1, but only the concomitant blocking on IL-4 and IL-13 can eliminate the induced Ym protein expression in BAL (10).

In addition, lipopolysaccharide (LPS) could boost the Ym protein expression induced by IL-4 *in vitro*, while IFN-γ could diminish the influence of IL-4, even causing Ym protein undetectable (3, 37). Glucocorticoids like dexamethasone were reported to induce Ym1 through STAT6, which have a co-enhancement effect with IL-4 on Ym1 (35). Simvastatin, a lipid-lowering drug, was also found to intensify Ym1 expression dependent on IL-4R (co-receptor of IL-4 and IL-13) (42). *In vivo*, endogenous TGF-β was reported to upregulate IL-4Rα, giving rise to a significant enhancement to the M2 activation of microglia caused by IL-4, thus increasing the synthesis and secretion of Ym1 (39, 40). Interestingly, opposite to its effects *in vitro*, dexamethasone was found to counteract the influence of IL-4 and reduce Ym1 expression in the ovalbumin (OVA)-induced asthma model (43). A cytokine homolog *Bm* macrophage migration inhibitory factor (MIF)-1 generated by the helminth parasite *Brugia malayi* showed an upregulating effect on Ym1 expression, in which case, IL-4 or IL-5 was not necessary for the induction of Ym1, but it remained unclear whether type 2 cytokines like IL-13 were required (29).

Moreover, accumulating evidence has suggested that the activation of immuno-metabolic regulatory peroxisome proliferator-activated receptor (PPAR)γ facilitates Ym1 expression in a STAT6-dependent manner. 15dPGJ2 (a natural PPARγ ligand) (44) and rosiglitazone (a PPARγ agonist) (32, 45) were reported to contribute to the Ym1 upregulation, while GW9662 (a selective antagonist) significantly blunted IL-4-induced Ym1 expression (46). Further, ChIP analysis proved Ym1 as one of the direct target genes of PPARγ (47), and the integrin α_V_β_5_ may play a crucial role in PPARγ-induced Ym1 upregulation (48).

In general, it is prevalent to use Ym1 as one of the detecting markers of M2 Mφ (3), especially for M2a activation induced by IL-4/13 (49). Ym1 has also been proposed as a marker of alternative neutrophil (N2) polarization (32, 50). However, a recent study has found that the M2 phenotype was enhanced in *Chil3*-deficient mice, demonstrating that Ym1 may control or limit the M2 activation of Mφ (6). Ym1 protein was also found to be absorbed by wound healing Mφ in Stat6-deficient mice (33), which might suggest that there could be some loopholes in the application of Ym1 as a Mφ phenotype marker, and it is recommended to define the Mφ phenotype together with other markers. Besides, Ym1 was observed as a unique one with increased expression among STAT6-associated M2 markers when STAT6 expression was augmented by the inhibitor of heat shock protein B1 (HSPB1) (51). These facts indicate that Ym1 serves as a function performer rather than a marker and zoom in the question that what the exact role of Ym1 is in M2 and relevant progress.

## Role of Ym1 in diseases

### Allergic lung inflammation

**Figure 4:**
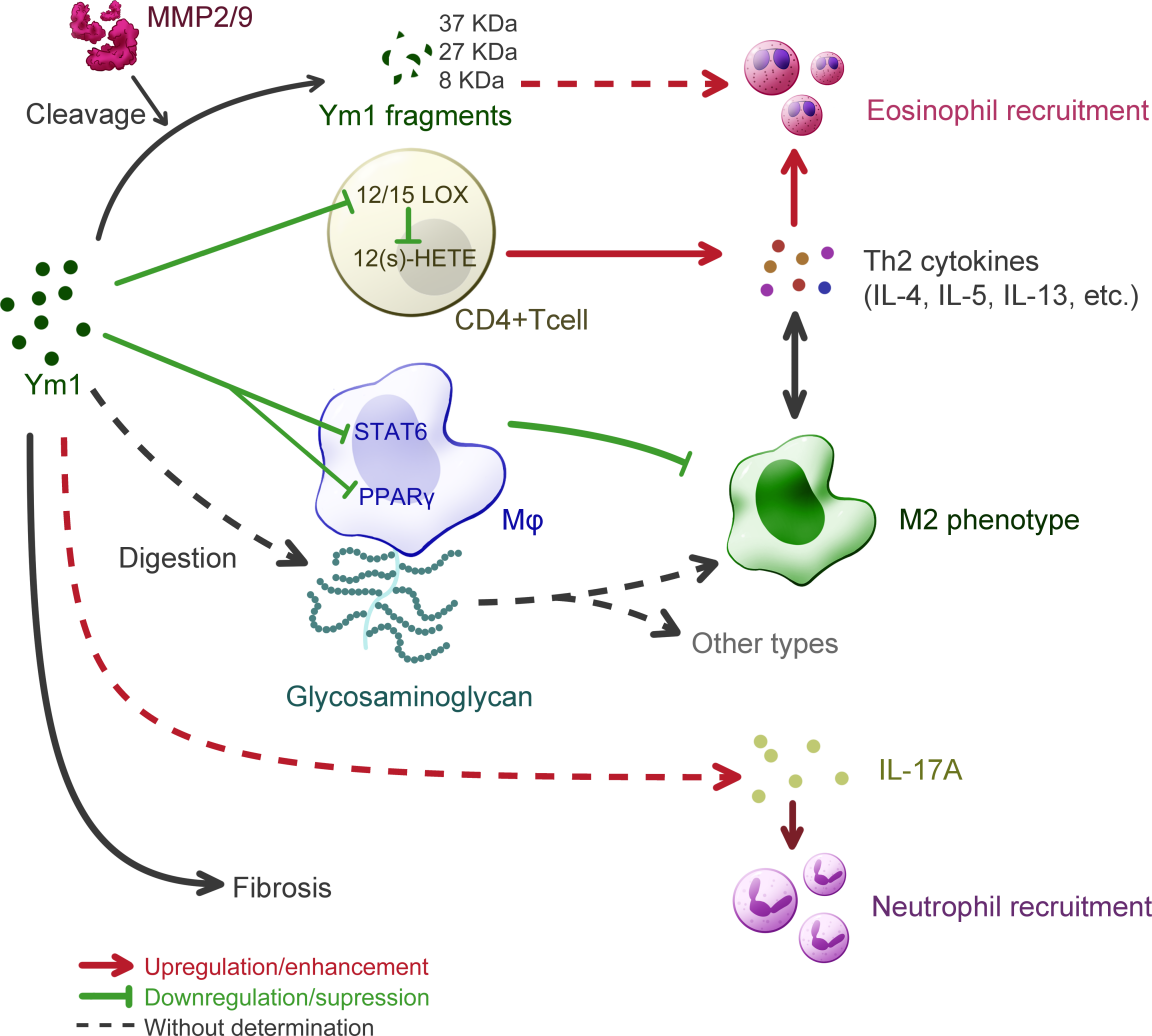
Understanding of Ym1 behavior in allergic lung inflammation in mice. The overall effect of Ym1 in Th2 allergic lung inflammation is intensifying eosinophil recruitment. Ym1 plays that in a combinatorial manner. Ym1 depresses 12/15(S)-lipoxygenase (12/15-LOX) in CD4^+^ T cells and its catalysate 12-hydroxyeicosatetraenoic acid (12(S)-HETE), leading to the rise of Th2 cytokines. And Ym1 limits M2 polarization by downregulating the activation of STAT6 and PPAR-γ in macrophages. It might also regulate phenotypes directly by digesting glycosaminoglycan on macrophage surface. Matrix metalloproteinase (MMP) 2/9 engage in this system as Ym1 catalytic crackers, whose products may help to eosinophil recruitment. Ym1 recruits neutrophils as well, which may depend on IL-17 responses and contribute to lung inflammation. At the end of allergic lung inflammation, Ym1 may affect fibrosis to some extent.

Allergic asthma is a chronic inflammatory disease of the lower respiratory tract, clinically leading to manifestations as recurrent wheezing, dyspnea, chest tightness and paroxysmal cough. Its pathophysiological features are inflammatory responses such as increased IgE synthesis, airway hyperresponsiveness, mucus hypersecretion and airway remodeling. The similar airway inflammation in lung can be modeled in mice challenged by some typical protein antigens like OVA or certain allergens like house dust mite (52). Recent studies have shown that the mechanisms driving the development of mild and severe asthma are different (53). Patients with mild and moderate asthma present a typical response, that is, helper T cell type 2 (Th2) inflammation, mediated by cytokines such as IL-4, IL-5 and IL-13, and eosinophilia (54). In contrast, patients with severe asthma could present a low Th2 and a high Th17 response, accompanied by neutrophil inflammation in airway (55). It was also found in mouse model that hyperresponsiveness could be induced without Th2 response but with increased IL-17 expression (56). Importantly, Ym1 has been found to be involved in the airway inflammation model in mice, and may lay effects on both types of mechanisms as shown in **Figure 4**.

On the one hand, Ym1 can intensify Th2 inflammatory response. The study on *Chil3*-deficient mice supports the effect of Ym1 on Th2 cytokines conclusively, as a fall was detected in the expression of IL-4 and IL-5 in *Chil3*-deficient mice during OVA induced pulmonary inflammation (6). This immunoregulatory effect of Ym1 was realized by M2 Mφ in disease. In human body, although the role of M2 Mφ has not been determined in asthma, it has been confirmed that the function of AMs in asthmatic patients is different from that of normal people (57). In the mouse model of allergic lung inflammation, Mφ tend to the M2 phenotype, which is considered unnecessary and should be inhibited by the body (58). Some studies, however, believe that the immune molecules on the M2 surface can mediate the uptake and clearance of allergens and control the development and severity of allergic inflammation, thus serving as protectors (59). Additionally, Ym1 or M2 could be unnecessary in the development of allergic airway disease, as IL-4Rα-impaired mice were still found classic airway inflammation in histologic pathology with decreased level of Ym1 and other M2 markers (60). Considerable research has been devoted to exploring the role of M2 Mφ in allergic lung inflammation with the detection of Ym1 as a marker, rather less attention has been paid to that whether Ym1 itself influenced these models. It was found that *Chil3*-deficiency could enhance the alternative activation of Mφ by regulating the activation of STAT6 and PPARγ pathways to alleviate pulmonary inflammation (6). Besides, Ym1 might also influence Mφ more directly due to its weak β-*N*-acetylglucosaminidase activity, which means a possibility to contribute to the digestion of glycosaminoglycans (5). Ym1 was presumed to involve in fine-tuning at the level of HS in Mφ, thereby affecting the activation of Mφ (49). DCs have also been demonstrated to generate Ym protein in response to IL-13 in the OVA-induced respiratory allergy, in which case Ym protein downregulated the activity of 12/15(S)-lipoxygenase (12/15-LOX) and the following products, 12-hydroxyeicosatetraenoic acid (12(S)-HETE), thereby enhancing the ability of CD4^+^ T cells to produce Th2 cytokines such as IL-5, IL-13, etc (61).

In addition, Ym1 may recruit eosinophils with the participation of matrix metalloproteinase (MMP) family. It was proposed that Ym1 protein might be modified or cleaved by MMPs to participate in the chemotaxis of eosinophils (62). In the complete *Aspergillus* allergen (CAA)-induced mouse model, multiple cleavage fragments of Ym1 protein were observed in BALF of wild-type mice, while more complete Ym1 protein in that of MMP2 and MMP9 double null (MMP2/9^-/-^) mice, and the number of eosinophils was also reduced. Ym1 was also proven to be the substrate of MMP2/9 and products would be 37 kDa, ~27 kDa and ~8 kDa peptide fragments. As MMPs are reported to clear allergic inflammatory cells in the lung by hydrolyzing protein, and MMP2 and MMP9 could also regulate the activity of Th2 chemokine, Ym1 may play a role in linking MMPs and eosinophils and participate in regulating the migration of allergic inflammatory cells to the pulmonary vesicle. By far, however, this view still lacks direct evidence.

On the other hand, in recent years, considerable literature has grown up around the role of IL-17 and neutrophils in allergic lung inflammation, and the influence of Ym1 is worthy of more attention. Ym1 may influence the recruitment of neutrophils by regulating IL-17 produced by γδT cells, which plays a key role in neutrophil-mediated defensive immunity (7, 63). And after anti-Ym1 antibody treatment, the number and proportion of neutrophils in the lung were decreased, and the expression of IL-17A and IL-17A target genes were also reduced (7). But in this case, eosinophilia, goblet cell proliferation and apnea enhancement did not show significant difference, so that further investigation is needed to prove the role of Ym1 in IL-17 related allergic inflammation in lung. In addition, in most models of airway inflammation for Ym1 study, mice were sensitized by OVA. LPS challenge, however, was suggested to realize the pathological state with strong Th17 response and modest Th2 response (64), thus might be a worth tool for studying the relationship between IL-17 and Ym1 in allergic inflammation, which has not gained enough attention.

Fibrosis is subject to Th2 and Th17 responses at the end stage of chronic inflammation like asthma, where M2 plays a vital role (65). Although no evidence has showed direct connection between Ym1 and fibrosis, J2, a pulmonary fibrosis suppressor, was found to upregulate Ym1 expression of M2 in the anti-fibrosis progress (51), which elucidated a possibility that Ym1 alleviates pulmonary fibrosis.

### Parasitic infection

Anti-parasitic immunity is characterized by eosinophilia and Th2 cytokines. In most anti-parasitic response studies in mice, Ym1 is regarded as a bridge between M2a cells and eosinophils, that is, Ym1 is secreted by Mφ activated by parasite antigens, and then participates in the recruitment of eosinophils to the injury site. For example, Bm-MIF-1 secreted by *Brugia malayi* was found involved in activating Mφ, inducing the upregulation of Ym1 expression, and cooperating with IL-5 to recruit eosinophils in a manner that is partially dependent on IL-4 (29). In another case, the larvae of *A.cantonensis* breaking into brain, was reported to induce M2 polarization of microglia and infiltrating Mφ within the CNS which then synthesized and secreted Ym1 in large quantities, accompanied by an increase in eosinophils (30). This idea was reinforced by an earlier study which confirmed the direct chemotactic effect of Ym on eosinophils *in vitro* (18). In this study recombinant Ym protein was applied to the back of mice subcutaneously in parasitic settings, and the result was consistent as Ym protein caused an abundant local recruitment of eosinophils. Nevertheless, people have not yet clarified the specific mechanism by which Ym1 protein “recruits” eosinophils. In the peritonitis, the expression of Ym1 was not proposed as a precondition for the recruitment of eosinophils, which means a possible substitutability of Ym1 in mice (10). In addition, an overexpression model through plasmid transfection showed that the exogenous expression of Ym1 protein in the lungs led to a decrease in the number of eosinophils but an increase in neutrophils (7). The differences among above studies might resulted from different situation (overexpression and different inflammatory models), but it at least indicates that inflammatory microenvironment could convert Ym1’s chemoattractant state.

Interestingly, in terms of Th2 cytokine response, Ym1 can play diametrically opposite roles in different stages of parasite response. After injecting anti-Ym1 antibodies into the peritoneal cavity, mice infected with *N. brasiliensis* were found that blocking Ym1 in the early innate immune stage could reduce the amount of Th2 cytokines in mice; after the establishment of an adaptive type 2 response, blocking Ym1 did not inhibit their expression, but significantly increased the number of cells expressing these factors (4). Although the reason why the effect of Ym1 changes with the course of the immune response has not yet been discovered, it again supports the multifaceted function of Ym1 in different inflammatory microenvironment.

In recent years, the role of neutrophils in the immune response against parasitic infection as well as its relationship with Ym1 has drawn researchers’ attention. Similar to eosinophils, as the “forerunner” of innate immunity, neutrophils also play a dual role in anti-parasitic immunity besides phagocytosis and initiation of Th2 response, and need to be contained in the later stage for inflammation resolution and tissue repair (66). In *N. brasiliensis*-infected mice, it was found that IL-17 and neutrophilic inflammation induced by Ym1 could impair parasite survival but at the cost of enhanced lung injury (7). This result suggested that Ym1 could be cursed blessing as well, and showed a possibility of Ym1 in the recruitment of neutrophils through regulating IL-17 production.

### Autoimmune diseases

Autoimmune diseases are a pathophysiological state, wherein the immune responses are directed against and damage the body’s own tissues, such as rheumatoid arthritis (RA) and psoriasis (Ps). Ym1 had been positionally identified to be associated with autoimmune arthritis, using collagen-induced arthritis (CIA) mouse model (67, 68). Later, a protective effect due to Ym1 deficiency using Ym1 congenic mice absent in Ym1 expression (6), was confirmed in collagen antibody induced arthritis (CAIA) model and mannan induced Ps model, both of which are Mφ dependent, and adaptive immune independent (69–71). The study also discovered that Ym1 protein i.n. supplement could reverse this effect in mannan induced Ps model of Ym1-low-expression mice, while AM depletion attenuated the disease. These results proved that Ym1 is one of the factors responsible for the development of skin and joint inflammation, and strongly suggested that Ym1 may participate in the diseases through regulating Mφ and innate immunity. However, how the M2 regulation from Ym1 in lung can ripple through the systemic autoimmunity remains unclear.

As for autoimmune neuroinflammatory diseases, the experimental autoimmune encephalomyelitis (EAE), which models the pathology of multiple sclerosis (MS) in mice, is often used to study the corresponding molecular mechanisms and treatment strategies. It was clarified that Ym1 was able to activate epidermal growth factor receptor (EGFR) and affected the directional differentiation of endogenous neural stem cells (NSCs) through the CLPs-EGFR-Pyk2 pathway by using EAE model mice (8). Therefore, drugs targeting the CLPs-EGFR-Pyk2 signaling axis may be used to treat acute demyelinating diseases such as neuromyelitis optica and relapsing multiple sclerosis. However, whether there is a direct interaction between Ym1 and EGFR as well as the proteins involved in the subsequent cascade needs to be further confirmed at the molecular level.

### Nervous system diseases

**Figure 5:**
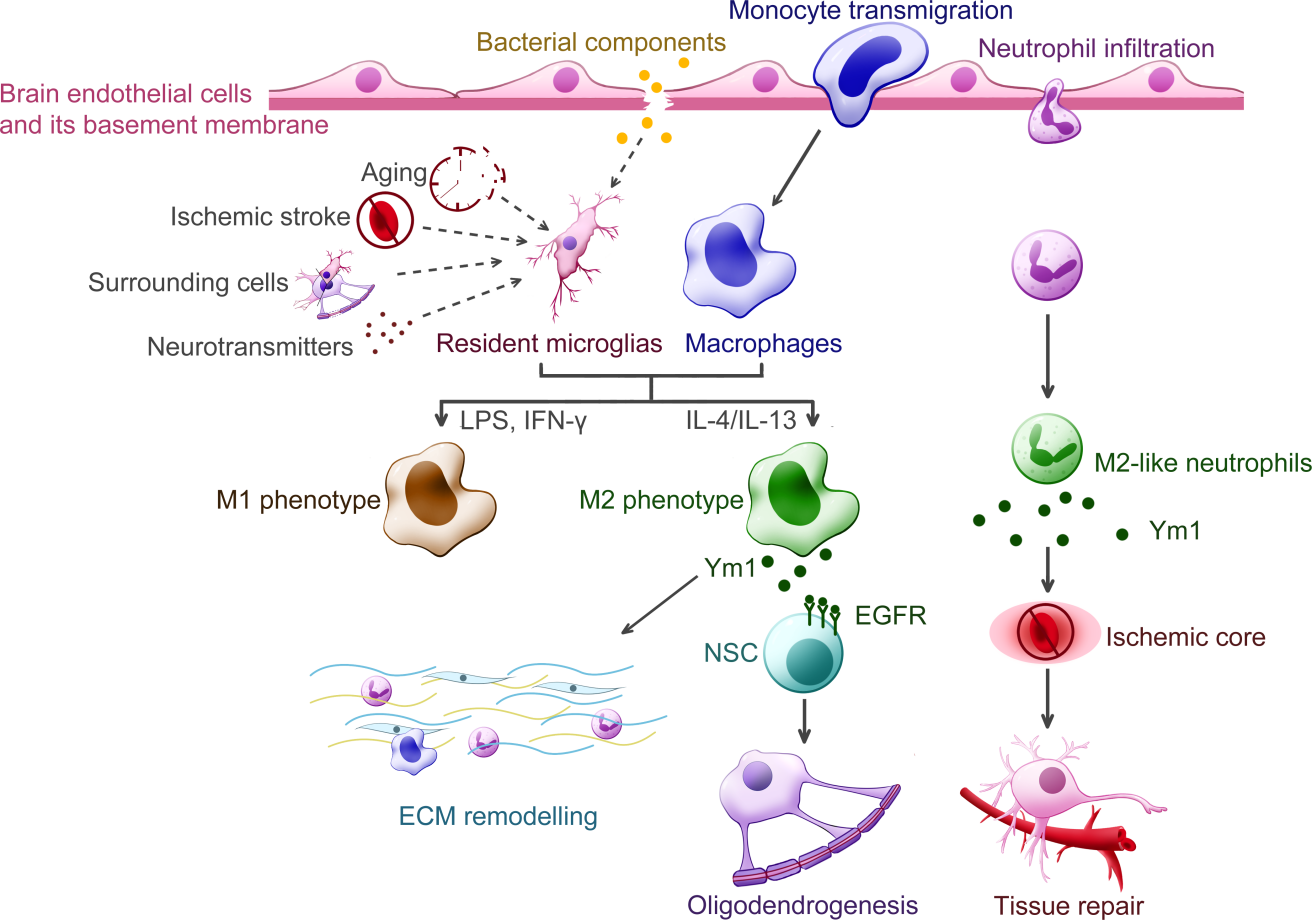
Putative functions of Ym1 in the central nervous system diseases. Various inflammatory stimuli promote the expression of Ym1, including bacterial infection, traumatic injury, ischemic stroke, aging and certain cytokines released from the surrounding cells. In the CNS, Ym1 is expressed and secreted by alternatively activated myeloid cells, including resident microglia, recruited macrophages, and even a subpopulation of neutrophils. Ym1 is proposed to facilitate extracellular matrix (ECM) remodeling for its binding specificity to particular components like heparan sulfate. In demyelinating diseases, Ym1 may bind to epidermal growth factor receptor (EGFR) of the neural stem cells (NSCs) and activate the Ym1-EGFR-Pyk2 pathway, leading to oligodendrogenesis. The Ym1-expressing neutrophils display increased ability to infiltrate the ischemic core and undergo phagocytosis, thereby contributing to inflammation resolution and neuroprotection.

In the context of nervous system diseases, most studies related to Ym1 fail to uncover its detailed function, where Ym1 usually takes the supporting role for marking M2. This is because the immune response compared with diseases occurring in other tissues and organs is more sophisticated, considering the difficulty of distinguishing resident microglia from recruited macrophages and outlining the temporal change in their polarization change under various stimuli (72). Meanwhile, it is still debatable whether Ym1 is a bona fide marker of M2-like cells or not, since Ym1 was observed to be upregulated with LPS exposure (a kind of M1 stimulation) alone (73). By far, Ym1 and its expression in traumatic injury and ischemic stroke is more recognizable. Therefore, the following section will focus primarily on these two types of diseases. **Figure 5** helped summarize the proposed functions of Ym1 from some key findings, which also included the aforementioned demyelinating diseases and bacterial infection.

Ischemic stroke is the primary type of cerebrovascular insult with high mortality risk. To unravel the mechanism of its immune response, Ym1 has greatly aided in the identification of microglia/Mφ polarization and a novel subpopulation of neutrophils (32). Researchers using the upregulated Ym1 expression to represent the neuroprotective cell phenotype, generally agreed on an increased M2-like state in the acute phase followed by an M1-like one in the subacute and chronic phase, though they didn’t specify the cellular origin of Ym1 until they started to analyze the cell specificity of each phenotype. Some pieces of research consistently suggested that recruited Mφ contributed remarkably to the gene expression of Ym1 at the early stage of ischemic stroke (74–76), whereas microglia was more pro-inflammatory and suppressive until about one week after stroke induction (76), with its *Chil3* promoter activity elevated for at least 14 days (31). In terms of Ym1’s function, the correlation between the upregulated *Chil3* mRNA and better post-stroke recovery was observed, including more neurovascular units (31), reduced infarct volume (75, 76), improved sensorimotor ability (74, 76), which could justify the protective role of Ym1. Besides, several intriguing findings of Ym1 are worth digging into, like its distribution was spatiotemporal (77) and its maximal expression and the time to peak didn’t seem to correlate with the lesion size (31). Hence, further investigations should monitor the actions of Ym1 protein over different regions and with different severity of stroke.

In response to traumatic injury, a series of orchestrated events occur in the peripheral nervous system (PNS) and CNS, where the activation and polarization of microglia/Mφ play a crucial role. Early studies found that Ym1 was heterogeneously expressed in penetrating brain injury and epileptic seizures (19), and it has been gradually used to mark M2-like microglia/Mφ, guiding us to learn the actions of immune cells in turn (78). Indeed, an increasing body of research has centered on the temporal profile of microglia/Mφ polarization, enabling us to determine which phenotype to enhance or suppress. Ym1, although typically represented the M2 phenotype (specifically the M2a (79) and M2c (72) marker) and upregulated both in the early inflammation stage and the later remodeling stage, has not been adequately studied yet. Ym1’s putative function in neuroinflammation and tissue repair is essentially based on its binding specificity to N-unsubstituted GlcN polymers and HS (2). Hence Ym1 is likely to antagonize inflammation by slowing down leukocyte adhesion and promote tissue repair by preventing HS from damage. In addition to its help in remyelination (8), only a few studies focused on its involvement in post-injury reactions. For instance, it was found that the accumulation of Ym1 protein within the injured olfactory epithelia was closely related to the inflammatory and healing process, with its level decreasing once tissue regeneration was achieved (24). In sum, to define Ym1’s function as neuroprotective still requires closer observations in its protein interactions with surrounding cells and tissues during the progression of neurotrauma.

## Crystallization of Ym1 *in vivo*

Crystals rarely spontaneously form in animals, but some proteins do spontaneously crystallize in animals under certain conditions. The typical example in the human body is Charcot-Leyden Crystals (CLCs), found by Ernst Viktor von Leyden in the sputum of patients with asthma in 1872. Subsequent studies found that the blood separated from patients with bronchial asthma was easy to form CLCs after lysis (80). Besides, eosinophilic crystals with similar morphology were observed in mice with lung cancer and mutant mice infected with *Pneumocystis carinii*. These crystals were finally confirmed to be Ym1, with similar morphology but different biochemical properties to CLCs (81).

*In vivo* crystallization of Ym1 was observed in many mouse models (see **Table 1**). In 1999, transgenic mice with over-expression of IL-13 had crystals similar to that of Charcot-Leyden crystals in the lungs, which were later confirmed as Ym1 (86, 90). In the same year, eosinophilic crystals with different shapes and sizes were observed in alveolar macrophages and multinucleated giant cells of a variety of immunodeficient mice, including Moth-eaten mice (viable motheaten mice, *me*^v^/*me*^v^), SPCTNFRIIFc transgenic mice, and CD40L-deficient mice spontaneously infected with *Pneumocystis carinii*. These crystals were distributed in the activated alveolar macrophages and dispersed in the lungs of young mice, while crystals located both intracellular and extracellular in the dying *me^v^/me^v^* mice and SPCTNFRIIFc transgenic mice (81). It is worth noting that 14 days after C57BL/6 mice are infected with *Cryptococcus neoformans*, the crystal structure was also visible in the lungs, which is similar to that of Ym1. Studies also found some crystals, whose composition was not strictly analyzed, formed at the edge of the polysaccharide membrane, and the progress was closely related to the deposition of intracellular polysaccharide CNPS, suggesting that bacterial capsular polysaccharides contributed to the protein enrichment of this crystal (87). Given that *Cryptococcus neoformans* also contain chitin and that Ym1 may be part of the host’s response to microorganisms containing chitin, this crystal is likely to be Ym1. Shortly afterward, Ym1 crystal was also found in p47phox ^-/-^ (p47phox subunit defect of NADPH oxidase) mice. This multifaceted crystal appeared outside the lung of mice older than two months and increased with age (5). Also, p47phox deficiency will cause macrophage dysfunction and eventually lead to progressive crystalline macrophage pneumonia (91). In tissue sections, the morphology of these Ym1 crystals has been described as intracellular fine needle-like crystals and flat, faceted crystals in BALF (5, 16, 81), thus providing new clues when similar crystals arise under some cases. In 2010, needle-like crystals were observed in the lung macrophages of the constructed heparanase-deficient mice (Hpse^-/-^), which were surrounded by membranes, suggesting that they are developed in capsule organelles such as lysosomes, endoplasmic reticulum or Golgi bodies. It is conclusively demonstrated that Ym1 is the crystal formation unit of Hpse^-/-^ alveolar macrophages, and heparanase regulates the accumulation and crystal formation of Ym1 in the airway (88). At the same time, in addition to various models or mice under specific conditions, several studies pointed out that Ym1 crystals or Ym1 protein accumulation existed in normal mouse lung macrophages (81, 88).

**Table 1:** Crystals formed in mice.

Mouse Models	Model Characters					Crystals		Reference
	Strain of Origin	Type of Gene Editing	Gene	Primary Effects	Diseases/Abnormal Effects	Location	Morphology	
***me*^v^/*me*^v^ mice(viable motheaten mice)**	C57BL/6J	Spontaneous mutation (recessive, single point)	*Ptpn6* gene, *motheaten (me)* locus on Chr6	SHP-1 protein tyrosine phosphatase activity deficiency	Severe autoimmune disease, premature death of pneumonitis, hematopoietic disorders, immune cell abnormality (hyperactivity of AM, lymphocytes, granulocytes in the lungs)	M2 and matrix in lungs	Long, rectangular crystals in tissues, flat crystals (10-μm^2^) and multifaceted crystals (20-120 μm) in BAL fluid of *me*^v^/*me*^v^ mice	(81–83)
**CD40L-deficient mice infected with *P. carinii***	C57BL/6C57BL/6NTac × Sv/129	Gene knockout	*CD40L* gene	CD40 ligand of activated T-cells deficiency (immunoglobulin isotype switching failure)	X-linked hyper-IgM syndrome, severe respiratory infection (cannot defend against *P. carinii*)	M2 in lungs		(81, 84)
**SPCTNFRIIFc transgenic mice**	C57BL/6NTac × Sv/129	Transgenic	Surfactant apoprotein C promotor/soluble TNF receptor p75 (type II)-Fc fusion protein	lung-specific protein sTNFRIIFc expression (a soluble TNF inhibitor)	Depression on TNF-α responses in lungs	M2 and occasionally matrix in lungs		(81, 85)
**mice with over-expression of IL-13**	CBA × C57BL/6	Transgenic	Clara cell 10-kDa protein (CC10) promoter/IL-13	IL-13 over-expression in airway	Asthma-like inflammatory responses in lungs	Eosinophils and AM in alveoli and occasionally in airways	Needle-like crystals	(86)
**C57BL/6 infected with *C. neoformans***	C57BL/6	–	–	–	Eosinophilic pneumonia (immune responses to microorganisms containing chitin)	Mφ-originated large multinucleated cells in lungs	Needle-like crystals (protruded through the membrane of some cells)*without strict determination of Ym1	(87)
**p47*^phox^* ^-^/^-^ mice**	129	Gene knockout	*p47^phox^* gene	p47phox subunit defect of NADPH oxidase in phagocytes	Chronic granulomatous disease	Lung matrix in aged mice (related to giant cells and Mφ)Bile ductsSpontaneous skin abscesses	Multifaceted crystals (10-100 μm)	(5)
***Hpse^-^/^-^* transgenic mice**	C57BL/6J	Gene knockout	*Heparanase* gene	Heparanase-deficient	Normally no major abnormalities	AM	Needle-like crystals	(88)
**ddY mice**	ddY	–	*-*	–	Spontaneous IgA nephropathy	Mφ in bone morrow (frequently with several erythroblasts)	Needle-like crystals*without strict determination of Ym1	(16, 89)

AM, alveolar macrophages; M2, alternatively activated macrophages.

In line with the tissue expression of Ym1 protein, Ym1 crystals were primarily found in the lungs, while they could also present outside the lungs. Membrane-encapsulated needle Ym1-immunoreactive crystals have been detected in macrophages in the bone marrow of ddY mice with spontaneous IgA nephropathy. The study also found that Ym1 was produced mainly by immature neutrophils and Ym1 may be phagocytosed by macrophages after forming crystals outside the cells, or directly absorbed by macrophages and crystallized in the cytoplasm (16).

In the above studies, there seems to be no clear relationship between the crystallization of Ym1 protein, but when crystals appear, Ym1 tends to show a state of high expression or abnormal protein degradation, resulting in the accumulation of Ym1 protein. At the same time, environmental factors are conducive to the formation of crystals. The high expression of Ym1 may be related to the function of Ym1 or the function of its ancestral genes (such as interaction with heparin, chitin and other substances). The pathological changes of tissues under different diseases may also provide similar environments for Ym1 crystallization (such as pH), which may be a new idea to study the causes of crystals.

Ym1 crystallization is a strong signal of lung inflammation and injury. As mentioned above, after purifying and identifying the Ym1 protein crystal in the BALF of me^v^/me^v^ mice, plenty of eosinophils were also observed in lungs (81). Collectively, Ym1 crystal was considered to be a reflection of the response to severe parasitic eosinophilic pneumonia. Besides, the formation of Ym1 crystal itself could also damage cell membrane mechanically and lead to cell death (87). Bronchial epithelial rupture directly leads to lung injury, and macrophage rupture death interferes with host defense mechanisms and causes persistent infection. Some studies suggested that Ym1 crystal might directly activate inflammatory bodies *in vivo*, resulting in lung injury (7). However, similar to CLCs in human body, relevant studies usually only observed the presence of Ym1 crystals in severe inflammatory environments. The role of crystals remains to be dug out.

## Discussion

In sum, Ym1 is expressed or upregulated under various pathological conditions, particularly the lung diseases. As a traditional M2 marker, Ym1 itself has not received adequate attention, given that most relevant studies focused on the Ym1-producing cells, including macrophages and microglia. However, we could comb out some intriguing clues to Ym1’s functions from previous literature resources. Current models demonstrate that the role of Ym1, albeit pleiotropic and dynamic, lays parallels between allergic lung inflammation and pulmonary parasite infection. Ym1 participates in these inflammatory responses generally in two ways, the modulation of Mφ polarization and the recruitment of eosinophils and neutrophils. And in both diseases, Ym1 generally shows association with two trends, the enhancement of Th2 response and IL-17 production, and the latter is gathering more attention. However, it is worth-noting that Ym1 displays time-dependent function in tissue repair and inflammation resolution. Ym1 not only promotes reparative Th2 response in the early phase of inflammation (34), but can reduce IL-5/IL-13 expression and regulate Th2 balance once the repair initiate (4). For skin and joint autoimmune inflammation, Ym1 contributes to its development through innate immunity, especially M2. In respect to the nervous system diseases, although existing research hardly distinguishes the cellular origins of Ym1, it generally agrees that Ym1s upregulation correlates with improved prognosis in most cases. A few Ym1-centered studies attempted to map the possible signal pathways for oligodendrogenesis, and to understand the relationships of Ym1 protein accumulation with olfactory epithelium injury, but the follow-up research is still lacking.

Considering that abundant C/CLPs exist in human bodies, despite no real homologous gene for Ym1, research on Ym1 has significance in facilitating the understanding of human C/CLPs in diseases. In addition, Ym1 is one of the only proteins that can form crystals in mice. Ym1 crystals are still poorly unraveled, but further explorations may help decipher the confusing eosinophilic crystals in human bodies, like CLCs in lungs. Previous work has not furnished details on how Ym1 exerts influence on other immune mediators like Th2 and Th17 cytokines or on the hierarchy of their actions in inflammatory responses. Thus, figuring out the position of Ym1 on these interactive networks is beneficial for revealing disease pathogenesis and finding optimal treatment targets and strategies. Besides, the kinetics of Ym1 expression is rather complex, depending on the site, mode and severity of injury. And recent research has cast doubt on whether Ym1 is a bona fide M2 marker. Hence future work should give the expression patterns of Ym1 upon different stimuli sufficient consideration.

## Author contributions

QK, LL and YP drafted the original manuscript, and WZ and LM advised on the outline and revised the manuscript. All authors contributed to the article and approved the submitted version.

## Funding

This work is supported by the National Natural Science Foundation of China (82171724, 82171784 and 81970029), and College Students’ Innovative Entrepreneurial Training Plan Program, Ministry of Education, China (S202110698612).

## Acknowledgement

We thank Huaizhi Jing from Xi’an Jiaotong University for collecting part of the literatures.

## Conflict of interest

The authors declare that the research was conducted in the absence of any commercial or financial relationships that could be construed as a potential conflict of interest.

## Publisher’s note

All claims expressed in this article are solely those of the authors and do not necessarily represent those of their affiliated organizations, or those of the publisher, the editors and the reviewers. Any product that may be evaluated in this article, or claim that may be made by its manufacturer, is not guaranteed or endorsed by the publisher.
